# Characterizing a Key Player in Micro, but Not Small, RNA Interference

**DOI:** 10.1371/journal.pbio.0040238

**Published:** 2006-06-13

**Authors:** Richard Robinson

Translation repression by RNA interference (RNAi) has emerged in recent years as a ubiquitous, powerful, and precise method for the regulation of gene expression, both naturally and experimentally. In this process, a messenger RNA (mRNA), made from a gene and destined to be translated into a protein, is silenced by a molecule of “interfering” RNA.

At least two related processes fall under the heading of RNAi; in each, the interfering RNA is a small molecule (about 21 nucleotides long) that binds to a matching sequence on the mRNA. Exactly what happens next depends on how close the match is. Perfect complementarity (involving so-called small interfering RNAs, or siRNAs) results in mRNA degradation, while an imperfect match leads to silencing of the message without degradation.

In this latter, non-degrading, form of interference, which has been widely observed in organisms from plants to people, the imperfectly complementary RNA is called a microRNA (miRNA), and is part of a large complex called the RISC (RNA-induced silencing complex). Neither the full structure of the RISC nor the details of its function are known, but several components have been identified, including the cleaving enzyme Dicer and the argonaute proteins Ago1 and Ago2. In the cytoplasm, after RISC binds to target mRNA, it accumulates in so-called P-bodies—poorly characterized multi-protein complexes that contain, among other things, the protein RCK/p54. In a new study, Chia-ying Chu and Tariq Rana demonstrate that, through its direct interactions with the argonaute proteins, and its promotion of P-body formation, RCK/p54 plays a key role in miRNA-mediated, but not siRNA-mediated, translation repression.

The authors began by determining which P-body proteins can bind to which RISC proteins, using fluorescent tags to mark the various proteins. They found that Ago2 formed complexes with most of the known players. But they reasoned that the RNA scaffolding of the P-body likely causes many proteins to associate without actually binding to one another, and so, to rule out nonspecific effects, they digested the RNA out of the preparation. This left only RCK/p54 and Ago1 clinging to Ago2, indicating a true direct interaction. They next asked whether RCK/p54, first identified in P-bodies, was also a component of the active RISC. By purifying active complexes and analyzing their composition, they showed that it was—when a RISC was activated by the presence of a microRNA, it also contained RCK/p54.

When the authors suppressed expression of RCK/p54 (neatly enough, by using an siRNA against it), P-bodies were disrupted, and Ago2 dispersed throughout the cytoplasm, suggesting RCK/p54 is responsible for the localization of Ago2 in P-bodies. P-bodies could also be disrupted by suppressing another protein, Lsm1; when this occurred, though, RCK/p54 still associated with Ago2 and with the RISC, indicating that these interactions do not depend on the presence of P-bodies.

RCK/p54 depletion also provided the authors with another important insight: loss of RCK/p54 did not prevent siRNA-mediated RNA cleavage—the form of translation repression that depends on exact complementarity between an siRNA and mRNA. It did, however, lead to an increase in translation of known microRNA targets, consistent with a role for RCK/p54 in the second, non-degrading, form of translation repression, mediated by miRNA.

Taken together, these results suggest that RCK/p54 forms part of the RISC in association with the argonaute proteins; that its previously identified association with P-bodies is a consequence of its function in the RISC; and that it plays a key role in miRNA-mediated translation repression. These results not only shed light on this important and poorly understood system for controlling gene expression, but also, because RCK/p54 appears to play no role in siRNA-mediated repression, they lay the groundwork for developing a detailed understanding of the similarities and differences between these two important pathways to RNAi.

## 

**Figure:**
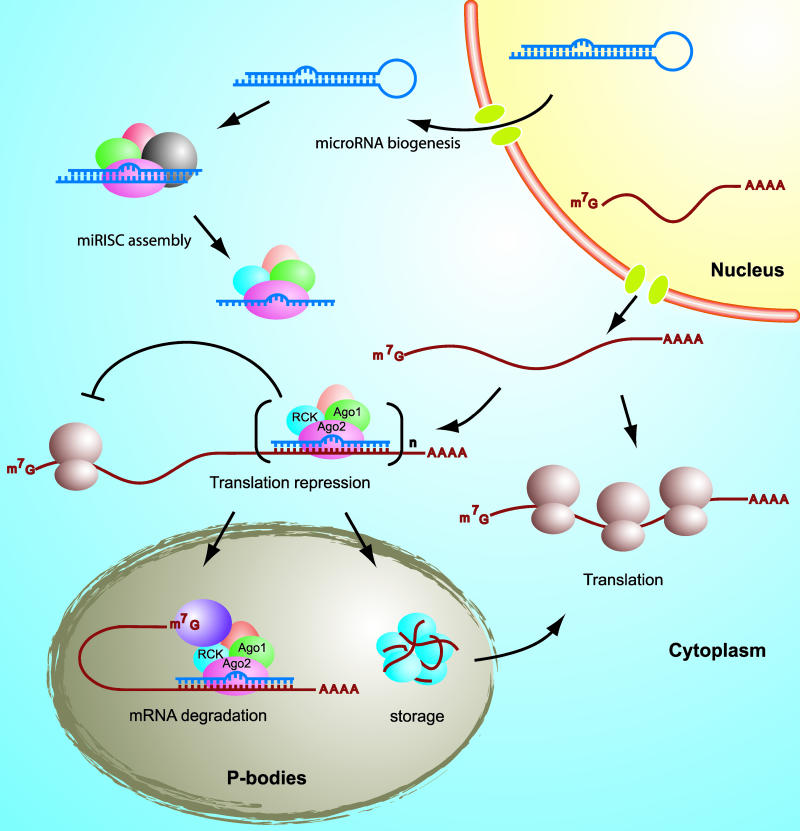
Mechanism of translational repression by human microRNAs.

